# Crystal structure of (*n*-but­yl)[2-(2,6-di­meth­oxy­phen­yl)-6-methyl­phen­yl](2-meth­oxy­phen­yl)phospho­nium chloride monohydrate

**DOI:** 10.1107/S2056989015024780

**Published:** 2016-01-13

**Authors:** Ge Feng, Alexander S. Filatov, Richard F. Jordan

**Affiliations:** a Department of Chemistry, the University of Chicago, 5735 South Ellis ave, Chicago, IL 60637, USA

**Keywords:** crystal structure, phospho­nium salt, hydrogen bonding

## Abstract

The title hydrated salt, C_26_H_32_O_3_P^+^·Cl^−^·H_2_O, contains four different substit­uents (H, alkyl, aryl, and biar­yl) on the P atom. The P—H hydrogen atom of the phospho­nium ion was located in a difference Fourier map and refined without imposing additional restraints. In the crystal, the Cl^−^ ions and water mol­ecules are linked by pairs of O_water_—H⋯Cl^−^ hydrogen bonds and further linked to the phospho­nium cation by P—H^+^⋯Cl^−^ and C_Ar/OMe_—H⋯O_water_ hydrogen bonds to form an infinite one-dimensional chain along the [010] direction.

## Abstract 2

In a phospho­nium chloride hydrated salt containing four different substituents (H, alkyl, aryl, and biar­yl) on the P atom, the Cl^−^ ions and water mol­ecules are linked by pairs of O_water_—H⋯Cl^−^ hydrogen bonds and further linked to the phospho­nium cation by P—H^+^⋯Cl^−^ and C_Ar/OMe_—H⋯O_water_ hydrogen bonds to form an infinite one-dimensional chain along the [010] direction.

## Chemical context   

Palladium(II) alkyl complexes that contain *ortho*-phosphino-arene­sulfonate ligands ([PO]^−^) exhibit unique behavior in olefin polymerization (Nakamura *et al.*, 2009 ▸; Ito & Nozaki, 2010 ▸; Nakamura *et al.*, 2013 ▸). One of the main drawbacks of traditional (PO)Pd alkyl catalysts is that they produce polyethyl­ene with only low-to-moderate mol­ecular weight (Drent *et al.*, 2002 ▸; Vela *et al.*, 2007 ▸). Studies have shown that incorporating bulky substituents on phospho­rous in the [PO]^−^ ligand is an effective strategy to increase the mol­ecular weight of the produced polymer (Skupov *et al.*, 2007 ▸; Shen & Jordan, 2009 ▸; Ota *et al.*, 2014 ▸). Therefore we were inter­ested in developing the new [PO]^−^ ligand **2** that contains bulky *P*-substituents (see Scheme). We attempted to prepare **2** by the reaction of (2-{2,6-(OMe)_2_-Ph}-6-Me-Ph)(2-OMe-Ph)PCl (**3**) with *in situ*-generated dili­thia­ted benzene­sulfonate to generate **2′**, followed by acidification with HCl. However, this procedure did not afford **2** but rather produced [(2-{2,6-(OMe)_2_-Ph}-6-Me-Ph)(2-OMe-Ph)(*n*-Bu)PH]Cl (**1**) in low yield after workup, which crystallizes as the monohydrate **1**·H_2_O (**I**). **1** likely formed by the reaction of **3** with the slight excess of *n*-BuLi present in the dili­thia­ted benzene­sulfonate solution. Here we report the crystal structure of **I**.
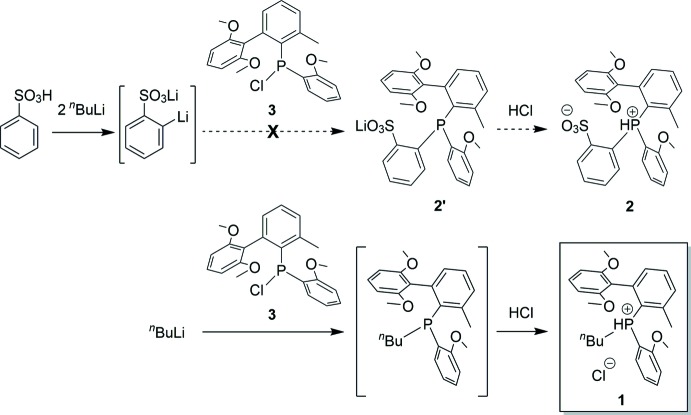


## Structural commentary   

**Figure 1:**
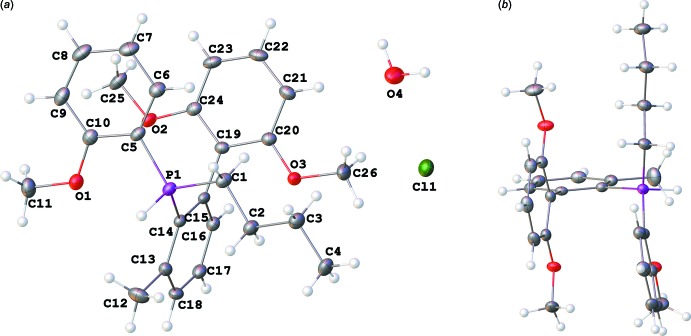
(*a*) The mol­ecular structure of **I** drawn with the 50% probability ellipsoids and showing the atom-labelling scheme. (*b*) A different view of **I** with H_2_O and Cl^−^ moieties omitted for clarity.

Crystals of **1**·H_2_O (**I**) suitable for X-ray diffraction analysis were obtained by recrystallization from wet CH_2_Cl_2_/Et_2_O (Fig. 1 ▸ *a*). The P—C bond lengths are almost equal for the alkyl, aryl, and biaryl substituents [1.7994 (14), 1.7824 (14), and 1.8043 (13) Å, respectively]. The C—P—H angles are also very similar [106.2 (7), 104.9 (7), and 107.5 (7)° for the alkyl, aryl, and biaryl substituents, respectively]. The aryl rings in the biaryl unit are essentially perpendicular to each other, with the angle between the mean planes passing through the six-membered rings being 88.60 (6)°. This conformation minimizes steric inter­actions between the *ortho*-meth­oxy groups and the *ortho*-hydrogens on the two rings. The mean planes passing through 2,6-di­meth­oxy­phenyl ring and the C-atoms of the 2-meth­oxy­phenyl and *n*-butyl groups are almost parallel to each other [the angle is 10.36 (5)°, Fig. 1 ▸ *b*]. The P—H hydrogen atom was located in a difference Fourier map and refined without additional restraints. The refined P—H bond length of 1.313 (16) Å is similar to those previously reported (Burke *et al.*, 2000 ▸, Zhu *et al.*, 2007 ▸, Wucher *et al.*, 2013 ▸).

## Supra­molecular features   

**Figure 2:**
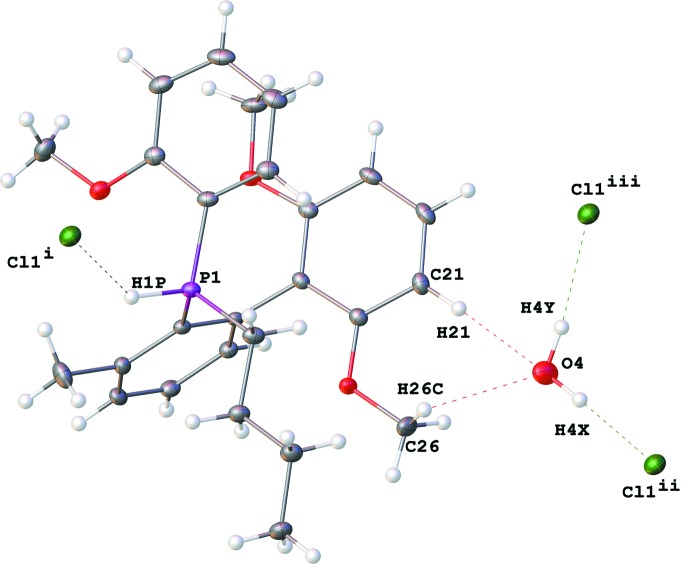
Hydrogen bonds in **I**. [Symmetry codes: (i) *x* − 1, *y* + 1, *z*; (ii) −*x* + 1, −*y*, −*z* + 1; (iii) *x* − 1, *y*, *z*.]

**Figure 3:**
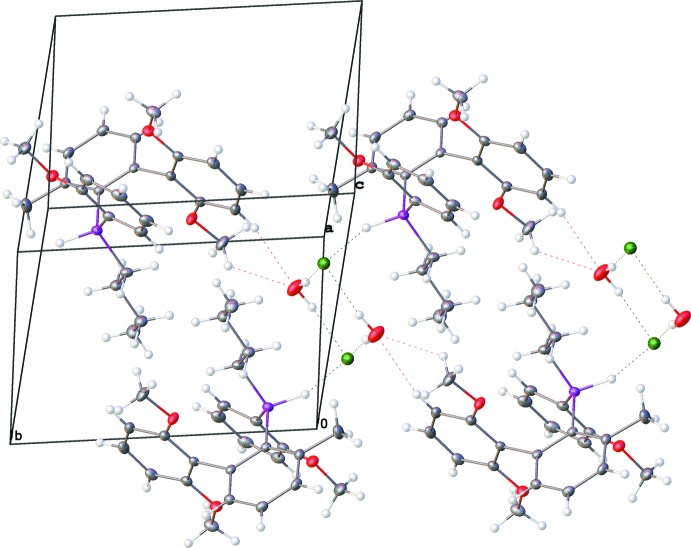
A fragment of the crystal packing of **I**.

The P—H^+^, Cl^−^, and water mol­ecule are involved in inter­molecular hydrogen bonding (Fig. 2 ▸, Table 1 ▸). Two Cl^−^ ions and two water mol­ecules form a rhombus (Fig. 3 ▸) in which the O⋯Cl distances are almost equal [3.1717 (13) and 3.1841 (13) Å]. The Cl^−^ ions are further engaged in P—H^+^⋯Cl^−^ hydrogen bonds [2.523 (16) Å], and the water mol­ecules are also involved in C_Ar/OMe_—H⋯O_water_ contacts [2.243 (16) and 2.254 (16) Å], forming infinite chains along the [010] direction (Fig. 3 ▸). The involvement of the P—H hydrogen atom in hydrogen bonding stands in contrast to what has been observed in some related structures. For example, in the structures of tri­phenyl­phospho­nium perchlorate (Zhu *et al.*, 2007 ▸) and tris­(*ortho*-tol­yl)phospho­nium tetra­chloro­borate (Burke *et al.*, 2000 ▸), there is no evidence for involvement of the P—H hydrogen atom in hydrogen bonding.

## Database survey   

A search of the Cambridge Structural Database (CSD, Version 5.36, last update May 2015; Groom & Allen, 2014 ▸) revealed that structures of phospho­nium salts having different alk­yl/ar­yl/biaryl substituents on phospho­rous are rare [CSD refcodes: BZMNPB (Böhme *et al.*, 1975 ▸), EDOSOF (Schiemenz *et al.*, 2002 ▸), SUXFUN (Dziuba *et al.*, 2010 ▸)]. To the best of our knowledge **I** is the first example of a crystallographically characterized phospho­nium salt having four different substituents at phospho­rous. Moreover, there are only three other examples of structures of protonated phospho­nium ar­yl/biaryl salts [CSD refcodes: WEMSIQ (Carre *et al.*, 1997 ▸), OCOWUY (Karaçar *et al.*, 2001 ▸), TOMZIF (Wang *et al.*, 2008 ▸)].

## Synthesis and crystallization   

(2-{2,6-(OMe)_2_-Ph}-6-Me-Ph)(2-OMe-Ph)PCl (**3**) was synthesized by a modification of a previously reported procedure (Neuwald *et al.*, 2013 ▸). The reaction of **3** with *in situ*-generated dili­thia­ted benzene­sulfonate was attempted to synthesize **2′** (see Scheme). However ^31^P and ESI–MS of the reaction mixture showed that **2′** was not formed. The reaction mixture was acidified with aqueous HCl and extracted with Et_2_O. After removal of volatiles from the Et_2_O fraction under vacuum, a yellow oil and white crystals (low yield) were obtained. Recrystallization of the white crystals from wet CH_2_Cl_2_/Et_2_O yielded crystals of [(2-{2,6-(OMe)_2_-Ph}-6-Me-Ph)(2-OMe-Ph)(*n*-Bu)PH]Cl·H_2_O (**1**·H_2_O, **I**), which was identified by X-ray crystallography analysis.

## Refinement   

Crystal data, data collection and structure refinement details are summarized in Table 2 ▸. Most of the carbon-bound H atoms were included in idealized positions for structure factor calculations [C—H = 0.95–0.98 Å, *U*_iso_(H) set to 1.2–1.5*U*_eq_(C)]. The P*—*H hydrogen atom and the H atoms of the butyl group were located in a difference Fourier map and refined without additional restraints. The H atoms bound to oxygen atom O4 were also located in the difference Fourier map but were restrained to be at 0.96 Å from O4 (within 0.02 Å) with their thermal parameters set to 1.5*U*_eq_ of O4.

## Supplementary Material

Crystal structure: contains datablock(s) I. DOI: 10.1107/S2056989015024780/hg5467sup1.cif

Structure factors: contains datablock(s) I. DOI: 10.1107/S2056989015024780/hg5467Isup2.hkl

Supporting information file. DOI: 10.1107/S2056989015024780/hg5467Isup3.cdx

Supporting information file. DOI: 10.1107/S2056989015024780/hg5467Isup4.cml

CCDC reference: 1444199

Additional supporting information:  crystallographic information; 3D view; checkCIF report

## Figures and Tables

**Table 1:** Hydrogen-bond geometry (Å, °)

*D*—H⋯*A*	*D*—H	H⋯*A*	*D*⋯*A*	*D*—H⋯*A*
P1—H1*P*⋯Cl1^i^	1.313 (16)	2.523 (16)	3.5798 (5)	135.5 (10)
C21—H21⋯O4	0.95	2.53	3.4594 (19)	167
C26—H26*C*⋯O4	0.98	2.53	3.2250 (19)	128
O4—H4*X*⋯Cl1^ii^	0.93 (2)	2.24 (2)	3.1717 (13)	173 (2)
O4—H4*Y*⋯Cl1^iii^	0.94 (2)	2.25 (2)	3.1841 (13)	173 (2)

Symmetry codes: (i) 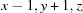; (ii) 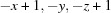; (iii) 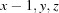.

**Table 2:** Experimental details

Crystal data	
Chemical formula	C_26_H_32_O_3_P^+^·Cl^−^·H_2_O
*M*_r_	476.95
Crystal system, space group	Triclinic, *P* 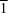
Temperature (K)	100
*a*, *b*, *c* (Å)	9.6920 (6), 10.2790 (6), 12.4154 (8)
α, β, γ (°)	96.836 (2), 98.481 (2), 94.188 (2)
*V* (Å^3^)	1209.47 (13)
*Z*	2
Radiation type	Mo *K*α
μ (mm^−1^)	0.25
Crystal size (mm)	0.22 × 0.15 × 0.14

Data collection	
Diffractometer	Bruker D8 Venture PHOTON 100 CMOS
Absorption correction	Numerical (*SADABS*; Bruker, 2014 ▸)
*T*_min_, *T*_max_	0.959, 0.987
No. of measured, independent and observed [*I* > 2σ(*I*)] reflections	33225, 6228, 5241
*R*_int_	0.028
(sin θ/λ)_max_ (Å^−1^)	0.677

Refinement	
*R*[*F* ^2^ > 2σ(*F* ^2^)], *wR*(*F* ^2^), *S*	0.038, 0.096, 1.04
No. of reflections	6228
No. of parameters	339
No. of restraints	2
H-atom treatment	H atoms treated by a mixture of independent and constrained refinement
Δρ_max_, Δρ_min_ (e Å^−3^)	0.51, −0.18

Computer programs: *APEX2* and *SAINT* (Bruker, 2012), *SHELXT2014* (Sheldrick, 2015*a* ▸), *SHELXL2014* (Sheldrick, 2015*b* ▸), *OLEX2* (Dolomanov *et al.*, 2009 ▸), *Mercury* (Macrae *et al.*, 2008 ▸), and *publCIF* (Westrip, 2010 ▸).

## supplementary crystallographic information

### Crystal data

**Table:**

C_26_H_32_O_3_P^+^·Cl^−^·H_2_O	*Z* = 2
*M**_r_* = 476.95	*F*(000) = 508
Triclinic, *P*1	*D*_x_ = 1.310 Mg m^−^^3^
*a* = 9.6920 (6) Å	Mo *K*α radiation, λ = 0.71073 Å
*b* = 10.2790 (6) Å	Cell parameters from 9958 reflections
*c* = 12.4154 (8) Å	θ = 2.4–28.7°
α = 96.836 (2)°	µ = 0.25 mm^−^^1^
β = 98.481 (2)°	*T* = 100 K
γ = 94.188 (2)°	Block, colorless
*V* = 1209.47 (13) Å^3^	0.22 × 0.15 × 0.14 mm

### Data collection

**Table:**

Bruker D8 Venture PHOTON 100 CMOS diffractometer	6228 independent reflections
Radiation source: INCOATEC IµS micro-focus source	5241 reflections with *I* > 2σ(*I*)
Mirrors monochromator	*R*_int_ = 0.028
Detector resolution: 10.4167 pixels mm^-1^	θ_max_ = 28.8°, θ_min_ = 2.1°
ω and phi scans	*h* = −13→13
Absorption correction: numerical (*SADABS*; Bruker, 2014)	*k* = −13→13
*T*_min_ = 0.959, *T*_max_ = 0.987	*l* = −16→16
33225 measured reflections	

### Refinement

**Table:**

Refinement on *F*^2^	Primary atom site location: dual
Least-squares matrix: full	Secondary atom site location: difference Fourier map
*R*[*F*^2^ > 2σ(*F*^2^)] = 0.038	Hydrogen site location: mixed
*wR*(*F*^2^) = 0.096	H atoms treated by a mixture of independent and constrained refinement
*S* = 1.04	*w* = 1/[σ^2^(*F*_o_^2^) + (0.0446*P*)^2^ + 0.6272*P*] where *P* = (*F*_o_^2^ + 2*F*_c_^2^)/3
6228 reflections	(Δ/σ)_max_ < 0.001
339 parameters	Δρ_max_ = 0.51 e Å^−^^3^
2 restraints	Δρ_min_ = −0.18 e Å^−^^3^

### Special details

**Table:**

Geometry. All e.s.d.'s (except the e.s.d. in the dihedral angle between two l.s. planes) are estimated using the full covariance matrix. The cell e.s.d.'s are taken into account individually in the estimation of e.s.d.'s in distances, angles and torsion angles; correlations between e.s.d.'s in cell parameters are only used when they are defined by crystal symmetry. An approximate (isotropic) treatment of cell e.s.d.'s is used for estimating e.s.d.'s involving l.s. planes.

### Fractional atomic coordinates and isotropic or equivalent isotropic displacement parameters (Å^2^)

**Table:**

	*x*	*y*	*z*	*U*_iso_*/*U*_eq_	
Cl1	0.99543 (4)	0.06475 (3)	0.71011 (3)	0.02320 (9)	
P1	0.22865 (3)	0.81030 (3)	0.70834 (3)	0.01537 (9)	
H1P	0.1976 (17)	0.9280 (16)	0.6876 (14)	0.021 (4)*	
O1	0.28474 (11)	0.98104 (10)	0.90415 (8)	0.0218 (2)	
O2	0.41677 (10)	0.67819 (10)	0.96813 (8)	0.0191 (2)	
O3	0.39185 (11)	0.49203 (9)	0.60356 (8)	0.0202 (2)	
C1	0.14332 (15)	0.69712 (15)	0.59177 (11)	0.0191 (3)	
H1A	0.1528 (17)	0.6081 (17)	0.6062 (14)	0.021 (4)*	
H1B	0.0437 (19)	0.7148 (17)	0.5838 (14)	0.025 (4)*	
C2	0.20590 (15)	0.71909 (15)	0.48809 (11)	0.0200 (3)	
H2A	0.3053 (18)	0.7053 (16)	0.4998 (14)	0.020 (4)*	
H2B	0.1977 (17)	0.8097 (17)	0.4745 (14)	0.021 (4)*	
C3	0.13321 (16)	0.62469 (15)	0.38878 (12)	0.0228 (3)	
H3A	0.035 (2)	0.6419 (17)	0.3743 (15)	0.028 (5)*	
H3B	0.1359 (19)	0.5340 (19)	0.4054 (15)	0.029 (5)*	
C4	0.20372 (19)	0.63906 (17)	0.28846 (13)	0.0266 (3)	
H4A	0.300 (2)	0.6220 (18)	0.3021 (15)	0.027 (5)*	
H4B	0.157 (2)	0.581 (2)	0.2247 (17)	0.038 (5)*	
H4C	0.201 (2)	0.725 (2)	0.2709 (16)	0.034 (5)*	
C5	0.15371 (14)	0.78501 (14)	0.82781 (11)	0.0165 (3)	
C6	0.05961 (14)	0.67784 (15)	0.83277 (12)	0.0201 (3)	
H6	0.0367	0.6102	0.7721	0.024*	
C7	−0.00070 (15)	0.67025 (16)	0.92696 (13)	0.0239 (3)	
H7	−0.0642	0.5969	0.9314	0.029*	
C8	0.03227 (15)	0.77041 (16)	1.01452 (12)	0.0245 (3)	
H8	−0.0104	0.7652	1.0782	0.029*	
C9	0.12588 (15)	0.87774 (15)	1.01127 (12)	0.0225 (3)	
H9	0.1471	0.9456	1.0718	0.027*	
C10	0.18838 (14)	0.88450 (14)	0.91790 (11)	0.0186 (3)	
C11	0.32790 (17)	1.08374 (15)	0.99388 (13)	0.0269 (3)	
H11A	0.2459	1.1266	1.0125	0.040*	
H11B	0.3947	1.1486	0.9728	0.040*	
H11C	0.3727	1.0463	1.0577	0.040*	
C12	0.43520 (17)	1.03920 (15)	0.66031 (14)	0.0288 (3)	
H12A	0.5106	1.1035	0.6504	0.043*	
H12B	0.3830	1.0780	0.7160	0.043*	
H12C	0.3718	1.0145	0.5905	0.043*	
C13	0.49738 (14)	0.91817 (13)	0.69739 (11)	0.0173 (3)	
C14	0.41672 (13)	0.81018 (13)	0.72460 (10)	0.0141 (2)	
C15	0.48062 (13)	0.70142 (12)	0.76063 (10)	0.0138 (2)	
C16	0.62572 (14)	0.70164 (13)	0.76972 (11)	0.0165 (3)	
H16	0.6703	0.6293	0.7952	0.020*	
C17	0.70582 (14)	0.80644 (14)	0.74193 (11)	0.0185 (3)	
H17	0.8045	0.8052	0.7476	0.022*	
C18	0.64171 (15)	0.91265 (14)	0.70595 (11)	0.0192 (3)	
H18	0.6974	0.9836	0.6866	0.023*	
C19	0.39855 (13)	0.58266 (13)	0.78584 (11)	0.0145 (3)	
C20	0.35655 (14)	0.47546 (13)	0.70350 (11)	0.0166 (3)	
C21	0.28231 (14)	0.36210 (13)	0.72404 (12)	0.0197 (3)	
H21	0.2515	0.2913	0.6671	0.024*	
C22	0.25470 (14)	0.35547 (14)	0.82964 (13)	0.0212 (3)	
H22	0.2044	0.2786	0.8446	0.025*	
C23	0.29803 (14)	0.45739 (14)	0.91409 (12)	0.0202 (3)	
H23	0.2791	0.4499	0.9861	0.024*	
C24	0.36994 (14)	0.57144 (13)	0.89184 (11)	0.0164 (3)	
C25	0.38278 (16)	0.67481 (16)	1.07612 (12)	0.0233 (3)	
H25A	0.2811	0.6580	1.0716	0.035*	
H25B	0.4148	0.7595	1.1211	0.035*	
H25C	0.4291	0.6046	1.1097	0.035*	
C26	0.36073 (17)	0.38149 (15)	0.51867 (12)	0.0267 (3)	
H26A	0.4082	0.3066	0.5429	0.040*	
H26B	0.3934	0.4048	0.4518	0.040*	
H26C	0.2593	0.3577	0.5034	0.040*	
O4	0.12871 (13)	0.13559 (12)	0.50244 (11)	0.0373 (3)	
H4X	0.097 (2)	0.072 (2)	0.4420 (16)	0.056*	
H4Y	0.085 (2)	0.108 (2)	0.5594 (16)	0.056*	

### Atomic displacement parameters (Å^2^)

**Table:**

	*U*^11^	*U*^22^	*U*^33^	*U*^12^	*U*^13^	*U*^23^
Cl1	0.02306 (18)	0.02389 (18)	0.02263 (18)	0.00422 (13)	0.00472 (13)	0.00038 (13)
P1	0.01430 (16)	0.01926 (17)	0.01294 (17)	0.00220 (13)	0.00277 (12)	0.00249 (13)
O1	0.0250 (5)	0.0225 (5)	0.0166 (5)	−0.0003 (4)	0.0035 (4)	−0.0008 (4)
O2	0.0213 (5)	0.0242 (5)	0.0124 (5)	0.0012 (4)	0.0050 (4)	0.0025 (4)
O3	0.0268 (5)	0.0180 (5)	0.0148 (5)	−0.0052 (4)	0.0061 (4)	−0.0008 (4)
C1	0.0161 (6)	0.0259 (7)	0.0145 (6)	0.0002 (5)	0.0012 (5)	0.0024 (5)
C2	0.0201 (7)	0.0246 (7)	0.0150 (7)	0.0006 (6)	0.0029 (5)	0.0020 (5)
C3	0.0250 (8)	0.0263 (8)	0.0167 (7)	0.0005 (6)	0.0029 (6)	0.0021 (6)
C4	0.0359 (9)	0.0280 (8)	0.0164 (7)	0.0028 (7)	0.0079 (6)	0.0002 (6)
C5	0.0138 (6)	0.0232 (7)	0.0138 (6)	0.0049 (5)	0.0038 (5)	0.0038 (5)
C6	0.0148 (6)	0.0265 (7)	0.0192 (7)	0.0026 (5)	0.0027 (5)	0.0034 (6)
C7	0.0148 (6)	0.0341 (8)	0.0250 (8)	0.0013 (6)	0.0059 (5)	0.0094 (6)
C8	0.0177 (7)	0.0401 (9)	0.0192 (7)	0.0092 (6)	0.0076 (5)	0.0086 (6)
C9	0.0211 (7)	0.0301 (8)	0.0172 (7)	0.0100 (6)	0.0041 (5)	0.0013 (6)
C10	0.0169 (6)	0.0223 (7)	0.0172 (7)	0.0058 (5)	0.0015 (5)	0.0042 (5)
C11	0.0322 (8)	0.0238 (7)	0.0219 (7)	0.0016 (6)	0.0004 (6)	−0.0036 (6)
C12	0.0285 (8)	0.0207 (7)	0.0391 (9)	0.0007 (6)	0.0052 (7)	0.0133 (7)
C13	0.0210 (7)	0.0156 (6)	0.0151 (6)	−0.0004 (5)	0.0032 (5)	0.0018 (5)
C14	0.0137 (6)	0.0167 (6)	0.0118 (6)	−0.0001 (5)	0.0030 (5)	0.0006 (5)
C15	0.0155 (6)	0.0155 (6)	0.0098 (6)	−0.0016 (5)	0.0031 (5)	0.0001 (5)
C16	0.0161 (6)	0.0180 (6)	0.0155 (6)	0.0026 (5)	0.0032 (5)	0.0013 (5)
C17	0.0151 (6)	0.0222 (7)	0.0176 (7)	−0.0010 (5)	0.0048 (5)	−0.0009 (5)
C18	0.0204 (7)	0.0184 (6)	0.0182 (7)	−0.0058 (5)	0.0058 (5)	0.0012 (5)
C19	0.0123 (6)	0.0161 (6)	0.0159 (6)	0.0013 (5)	0.0024 (5)	0.0047 (5)
C20	0.0146 (6)	0.0181 (6)	0.0173 (6)	0.0011 (5)	0.0026 (5)	0.0041 (5)
C21	0.0168 (6)	0.0163 (6)	0.0257 (7)	−0.0007 (5)	0.0021 (5)	0.0045 (5)
C22	0.0154 (6)	0.0210 (7)	0.0296 (8)	−0.0004 (5)	0.0056 (5)	0.0113 (6)
C23	0.0166 (6)	0.0268 (7)	0.0208 (7)	0.0035 (5)	0.0071 (5)	0.0114 (6)
C24	0.0134 (6)	0.0200 (6)	0.0171 (6)	0.0041 (5)	0.0035 (5)	0.0050 (5)
C25	0.0236 (7)	0.0348 (8)	0.0147 (7)	0.0091 (6)	0.0079 (5)	0.0059 (6)
C26	0.0329 (8)	0.0233 (7)	0.0209 (7)	−0.0081 (6)	0.0074 (6)	−0.0062 (6)
O4	0.0384 (7)	0.0375 (7)	0.0322 (7)	−0.0162 (5)	0.0104 (5)	−0.0042 (5)

### Geometric parameters (Å, º)

**Table:**

P1—C5	1.7824 (14)	C11—H11B	0.9800
P1—C1	1.7994 (14)	C11—H11C	0.9800
P1—C14	1.8043 (13)	C12—C13	1.512 (2)
P1—H1P	1.313 (16)	C12—H12A	0.9800
O1—C10	1.3557 (17)	C12—H12B	0.9800
O1—C11	1.4313 (17)	C12—H12C	0.9800
O2—C24	1.3639 (17)	C13—C18	1.3930 (19)
O2—C25	1.4307 (16)	C13—C14	1.4135 (18)
O3—C20	1.3611 (16)	C14—C15	1.4042 (18)
O3—C26	1.4367 (17)	C15—C16	1.3940 (18)
C1—C2	1.5347 (19)	C15—C19	1.4979 (17)
C1—H1A	0.962 (17)	C16—C17	1.3884 (19)
C1—H1B	0.988 (18)	C16—H16	0.9500
C2—C3	1.522 (2)	C17—C18	1.382 (2)
C2—H2A	0.976 (17)	C17—H17	0.9500
C2—H2B	0.973 (17)	C18—H18	0.9500
C3—C4	1.523 (2)	C19—C24	1.4005 (18)
C3—H3A	0.975 (19)	C19—C20	1.4030 (18)
C3—H3B	0.980 (19)	C20—C21	1.3939 (19)
C4—H4A	0.952 (19)	C21—C22	1.385 (2)
C4—H4B	0.96 (2)	C21—H21	0.9500
C4—H4C	0.94 (2)	C22—C23	1.385 (2)
C5—C6	1.391 (2)	C22—H22	0.9500
C5—C10	1.4058 (19)	C23—C24	1.3967 (19)
C6—C7	1.390 (2)	C23—H23	0.9500
C6—H6	0.9500	C25—H25A	0.9800
C7—C8	1.388 (2)	C25—H25B	0.9800
C7—H7	0.9500	C25—H25C	0.9800
C8—C9	1.385 (2)	C26—H26A	0.9800
C8—H8	0.9500	C26—H26B	0.9800
C9—C10	1.3913 (19)	C26—H26C	0.9800
C9—H9	0.9500	O4—H4X	0.933 (16)
C11—H11A	0.9800	O4—H4Y	0.935 (16)
			
C5—P1—C1	110.72 (7)	H11B—C11—H11C	109.5
C5—P1—C14	115.02 (6)	C13—C12—H12A	109.5
C1—P1—C14	111.73 (6)	C13—C12—H12B	109.5
C5—P1—H1P	104.9 (7)	H12A—C12—H12B	109.5
C1—P1—H1P	106.2 (7)	C13—C12—H12C	109.5
C14—P1—H1P	107.5 (7)	H12A—C12—H12C	109.5
C10—O1—C11	117.40 (11)	H12B—C12—H12C	109.5
C24—O2—C25	117.30 (11)	C18—C13—C14	118.04 (12)
C20—O3—C26	117.23 (11)	C18—C13—C12	118.55 (12)
C2—C1—P1	111.17 (10)	C14—C13—C12	123.41 (12)
C2—C1—H1A	109.2 (10)	C15—C14—C13	120.89 (12)
P1—C1—H1A	110.0 (10)	C15—C14—P1	119.76 (10)
C2—C1—H1B	111.4 (10)	C13—C14—P1	119.31 (10)
P1—C1—H1B	105.0 (10)	C16—C15—C14	118.88 (12)
H1A—C1—H1B	110.1 (14)	C16—C15—C19	118.49 (12)
C3—C2—C1	111.50 (12)	C14—C15—C19	122.59 (11)
C3—C2—H2A	108.6 (10)	C17—C16—C15	120.72 (13)
C1—C2—H2A	109.4 (10)	C17—C16—H16	119.6
C3—C2—H2B	110.2 (10)	C15—C16—H16	119.6
C1—C2—H2B	109.0 (10)	C18—C17—C16	119.89 (13)
H2A—C2—H2B	108.0 (14)	C18—C17—H17	120.1
C2—C3—C4	111.23 (12)	C16—C17—H17	120.1
C2—C3—H3A	108.7 (11)	C17—C18—C13	121.55 (12)
C4—C3—H3A	110.7 (11)	C17—C18—H18	119.2
C2—C3—H3B	109.5 (11)	C13—C18—H18	119.2
C4—C3—H3B	109.1 (11)	C24—C19—C20	118.43 (12)
H3A—C3—H3B	107.5 (15)	C24—C19—C15	121.77 (12)
C3—C4—H4A	111.1 (11)	C20—C19—C15	119.66 (11)
C3—C4—H4B	111.9 (12)	O3—C20—C21	123.42 (12)
H4A—C4—H4B	108.7 (16)	O3—C20—C19	115.07 (11)
C3—C4—H4C	110.1 (12)	C21—C20—C19	121.51 (13)
H4A—C4—H4C	107.5 (16)	C22—C21—C20	118.23 (13)
H4B—C4—H4C	107.3 (16)	C22—C21—H21	120.9
C6—C5—C10	120.16 (12)	C20—C21—H21	120.9
C6—C5—P1	123.45 (11)	C21—C22—C23	122.09 (13)
C10—C5—P1	116.29 (10)	C21—C22—H22	119.0
C7—C6—C5	119.61 (13)	C23—C22—H22	119.0
C7—C6—H6	120.2	C22—C23—C24	119.05 (13)
C5—C6—H6	120.2	C22—C23—H23	120.5
C8—C7—C6	119.68 (14)	C24—C23—H23	120.5
C8—C7—H7	120.2	O2—C24—C23	124.22 (12)
C6—C7—H7	120.2	O2—C24—C19	115.16 (12)
C9—C8—C7	121.58 (13)	C23—C24—C19	120.62 (13)
C9—C8—H8	119.2	O2—C25—H25A	109.5
C7—C8—H8	119.2	O2—C25—H25B	109.5
C8—C9—C10	118.89 (14)	H25A—C25—H25B	109.5
C8—C9—H9	120.6	O2—C25—H25C	109.5
C10—C9—H9	120.6	H25A—C25—H25C	109.5
O1—C10—C9	125.57 (13)	H25B—C25—H25C	109.5
O1—C10—C5	114.38 (12)	O3—C26—H26A	109.5
C9—C10—C5	120.05 (13)	O3—C26—H26B	109.5
O1—C11—H11A	109.5	H26A—C26—H26B	109.5
O1—C11—H11B	109.5	O3—C26—H26C	109.5
H11A—C11—H11B	109.5	H26A—C26—H26C	109.5
O1—C11—H11C	109.5	H26B—C26—H26C	109.5
H11A—C11—H11C	109.5	H4X—O4—H4Y	105 (2)
			
C5—P1—C1—C2	179.34 (10)	C13—C14—C15—C19	−177.42 (12)
C14—P1—C1—C2	−51.05 (12)	P1—C14—C15—C19	0.53 (17)
P1—C1—C2—C3	−179.38 (10)	C14—C15—C16—C17	−1.07 (19)
C1—C2—C3—C4	−175.19 (13)	C19—C15—C16—C17	176.72 (12)
C1—P1—C5—C6	10.21 (14)	C15—C16—C17—C18	0.7 (2)
C14—P1—C5—C6	−117.62 (12)	C16—C17—C18—C13	0.5 (2)
C1—P1—C5—C10	−166.26 (10)	C14—C13—C18—C17	−1.2 (2)
C14—P1—C5—C10	65.91 (12)	C12—C13—C18—C17	178.38 (13)
C10—C5—C6—C7	0.5 (2)	C16—C15—C19—C24	90.26 (16)
P1—C5—C6—C7	−175.84 (11)	C14—C15—C19—C24	−92.04 (16)
C5—C6—C7—C8	0.8 (2)	C16—C15—C19—C20	−85.45 (16)
C6—C7—C8—C9	−0.9 (2)	C14—C15—C19—C20	92.25 (16)
C7—C8—C9—C10	−0.2 (2)	C26—O3—C20—C21	−5.6 (2)
C11—O1—C10—C9	1.2 (2)	C26—O3—C20—C19	175.38 (12)
C11—O1—C10—C5	−178.31 (12)	C24—C19—C20—O3	−178.00 (11)
C8—C9—C10—O1	−177.92 (13)	C15—C19—C20—O3	−2.15 (18)
C8—C9—C10—C5	1.5 (2)	C24—C19—C20—C21	2.9 (2)
C6—C5—C10—O1	177.84 (12)	C15—C19—C20—C21	178.79 (12)
P1—C5—C10—O1	−5.57 (16)	O3—C20—C21—C22	178.79 (12)
C6—C5—C10—C9	−1.7 (2)	C19—C20—C21—C22	−2.2 (2)
P1—C5—C10—C9	174.93 (10)	C20—C21—C22—C23	0.2 (2)
C18—C13—C14—C15	0.86 (19)	C21—C22—C23—C24	1.0 (2)
C12—C13—C14—C15	−178.75 (13)	C25—O2—C24—C23	−3.54 (19)
C18—C13—C14—P1	−177.10 (10)	C25—O2—C24—C19	176.32 (11)
C12—C13—C14—P1	3.30 (18)	C22—C23—C24—O2	179.57 (12)
C5—P1—C14—C15	55.82 (12)	C22—C23—C24—C19	−0.3 (2)
C1—P1—C14—C15	−71.50 (12)	C20—C19—C24—O2	178.49 (11)
C5—P1—C14—C13	−126.20 (11)	C15—C19—C24—O2	2.72 (18)
C1—P1—C14—C13	106.48 (11)	C20—C19—C24—C23	−1.65 (19)
C13—C14—C15—C16	0.27 (19)	C15—C19—C24—C23	−177.41 (12)
P1—C14—C15—C16	178.22 (10)		

### Hydrogen-bond geometry (Å, º)

**Table:**

*D*—H···*A*	*D*—H	H···*A*	*D*···*A*	*D*—H···*A*
P1—H1*P*···Cl1^i^	1.313 (16)	2.523 (16)	3.5798 (5)	135.5 (10)
C21—H21···O4	0.95	2.53	3.4594 (19)	167
C26—H26*C*···O4	0.98	2.53	3.2250 (19)	128
O4—H4*X*···Cl1^ii^	0.93 (2)	2.24 (2)	3.1717 (13)	173 (2)
O4—H4*Y*···Cl1^iii^	0.94 (2)	2.25 (2)	3.1841 (13)	173 (2)

Symmetry codes: (i) *x*−1, *y*+1, *z*; (ii) −*x*+1, −*y*, −*z*+1; (iii) *x*−1, *y*, *z*.
